# Incidence and case fatality of aneurysmal subarachnoid hemorrhage admitted to hospital between 2008 and 2014 in Norway

**DOI:** 10.1007/s00701-020-04463-x

**Published:** 2020-06-30

**Authors:** Lise R. Øie, Ole Solheim, Paulina Majewska, Trond Nordseth, Tomm B. Müller, Sven M. Carlsen, Heidi Jensberg, Øyvind Salvesen, Sasha Gulati

**Affiliations:** 1 Department of Neurology, St. Olavs hospital, Trondheim University Hospital, 7006 Trondheim, Norway; 2 Department of Neuromedicine and Movement Science, Faculty of Medicine and Health Sciences, Norwegian University of Science and Technology (NTNU), Trondheim, Norway; 3 Department of Neurosurgery, St.Olavs hospital, Trondheim University Hospital, Trondheim, Norway; 4 Department of Anesthesiology, St. Olavs hospital, Trondheim University Hospital, Trondheim, Norway; 5 Department of Endocrinology, St. Olavs hospital, Trondheim University Hospital, Trondheim, Norway; 6 Department of Clinical and Molecular Medicine, Norwegian University of Science and Technology (NTNU), Trondheim, Norway; 7 Department of Health Registries, Directorate of Health, Trondheim, Norway; 8 Department of Public Health and General Practice, Norwegian University of Science and Technology (NTNU), Trondheim, Norway

**Keywords:** Subarachnoid hemorrhage, Incidence, Case fatality, Survival

## Abstract

**Background:**

To provide age- and sex-specific incidence and case fatality rates for non-traumatic aneurysmal subarachnoid hemorrhage (aSAH) in Norway. We also studied time trends in incidence and case fatality, as well as predictors of death following aSAH.

**Methods:**

A nationwide study using discharge data for patients admitted with aSAH between 2008 and 2014.

**Results:**

A total of 1732 patients with aSAH were included. The mean age was 60 years (SD 14) and 63% were females. Crude annual incidence was 5.7 per 100,000 person-years (95% CI 5.4–6.0) and was higher in females (6.3 per 100,000, 95% CI 5.9–6.7) compared with males (4.9 per 100,000, 95% CI 4.5–5.3). The annual decline in aSAH incidence was 3.2% per year (*p* = 0.007). The cumulative proportions of fatalities at days 30, 90, and 1 year were 22%, 25%, and 37%, respectively. The 30-day mortality rate did not change during the study period. Age (HR 0.7–2.2) and aneurysms in the posterior circulation (HR 1.7, 95% CI 1.3–2.3, *p* = 0.001) were associated with higher 30-day case fatality following aSAH, while aneurysm repair (HR 0.2, 95% CI 0.2–0.3, *p* < 0.001) was associated with lower risk.

**Conclusions:**

The incidence of aSAH declined in Norway between 2008 and 2014. Case fatality following aSAH continues to be high, and the 30-day mortality during the study period was unchanged. Increasing age and aneurysms in the posterior circulation were associated with increased risk of death within 30 days following aSAH.

## Introduction

Subarachnoid hemorrhage (SAH) from a ruptured aneurysm accounts for approximately 5% of all strokes and is a feared stroke subtype because of its high risk of poor functional outcome or death [2, 28]. SAH may occur at all ages but is more common between the 4th and 6th decade of life. A ruptured intracranial aneurysm is responsible for 85% of SAH cases [22], but the pathophysiology of aneurysm formation and rupture is still not fully understood. The type and size of the aneurysm, as well as female gender and higher age seem to be associated with the risk of rupture [34]. Despite some genetic predisposition, intracranial aneurysms are usually not congenital but develop throughout the course of life [39]. Thus, established and modifiable risk factors, such as hypertension, smoking, and alcohol abuse, remain most important to address in the prevention of aneurysmal SAH (aSAH) [8].

The overall incidence of stroke has declined during the past decades, attributed to the reduction of the proportion of people who smoke and better detection and treatment of hypertension through health education and behavioral change programs [1, 2, 6]. Smoking and hypertension are also risk factors for aSAH, but the decline in the incidence of aSAH over the past decades is relatively modest compared with that for stroke in general [2]. It has been suggested that there might be a lag time between the reduction of stroke risk factors and the effect on aSAH incidence [6]. An overall incidence rate of approximately 9.1 per 100,000 person-years of aSAH was reported in a systematic review from 2007, with doubled rates in Japan and Finland and far lower rates in South and Central America [2]. A more recent systematic review found a global incidence of 6.1 (95% CI 4.9–7.5) per 100,000 person-years [4]. Estimates of the incidence of true aSAH in the literature might be imprecise as many epidemiological studies on aSAH also include other causes, such as perimesencephalic SAH and arteriovenous malformations [17]. Another important reason for varying incidence rates in prior studies may be differences in study designs, i.e., different methods of case ascertainment, and different risk factor exposure and time periods [15]. Regarding survival after aSAH, there is quite consistent data on declining trends for case fatality following SAH [21, 23, 26, 28].

The current study was performed to investigate the national incidence and case fatality rates of aSAH in Norway for the years 2008 to 2014. The trends of annual aSAH rates were evaluated, as well as predictors of early death following aSAH.

## Methods

### Data source

#### The Norwegian health care system

Acute illness requiring hospital admission is treated free of charge by the Norwegian public health care system, and insurance policies do not influence the management of SAH. Only public hospitals provide inpatient health care to patients with SAH in Norway, and health authorities cover all inpatient treatment expenses.

#### Norwegian patient registry

The Norwegian patient registry (NPR) receives information regarding diagnoses from all patients receiving inpatient treatment by the Norwegian public specialist health care services. All discharge diagnoses are exclusively assigned by the physicians treating the patient and cannot later be altered. Based on NPR data we identified all patients hospitalized in Norway between 2008 and 2014 with a primary diagnosis of non-traumatic SAH (ICD-10 I60.0–I60.9). The validity of SAH diagnosis (ICD-10 I60.0–I60.9) in the NPR has been previously validated and found to have an overall confirmation rate of 95.3% [31, 40].

#### The Norwegian prescription database

The Norwegian prescription database contains information about all prescriptions dispensed in Norway since 2004 including the type of drug according to the Anatomical Therapeutic Chemical (ATC) classification. Diagnoses are registered for medications with reimbursement according to ICD-10 or version two of the International Classification of Primary Care [12]. Pharmacies are required to register each drug dispensed in the national prescription database, ensuring complete registration. Antithrombotic medications are only available at state-licensed pharmacies in Norway and dispensed to patients with a prescription from a physician. All filled prescriptions for oral formulations of antithrombotic medications were recorded including aspirin, dipyridamole, clopidogrel, prasugrel, ticagrelor, ticlodipine, warfarin, dabigatran, apixaban, rivaroxaban, dicumatrol, and phenylindandion. The use of oral antithrombotic drugs was registered as a dichotomous variable.

#### Study population

The number of inhabitants in Norway for each year during the study period was provided by Statistics Norway (Statistics Norway, Oslo, Norway). NPR identified patients aged 18 years or older with a first-time primary discharge code of non-traumatic SAH (I60.0–I60.9) within the same period. NPR and Norwegian Prescription database were linked on an individual level by a unique 11-digit personal identifier. Only patients with a valid personal identifier and a Norwegian residence permit were included in our study. Data from NPR were de-identified before provided to the study authors. The date of admission was used as the index date. Patients were followed until death or end of the study period, depending on what occurred first. In an attempt to decrease the likelihood of reporting inaccurate incidence rates for aSAH, we excluded SAH coded as non-aneurysmal SAH (I60.8-I60.9). The included aSAH were divided into hemorrhages of the 1) anterior circulation (I60.0-I60.3), 2) posterior circulation (I60.4-I60.5), and other intracranial locations (I60.6-I60.7). Due to Norwegian data privacy regulations, it was not possible to review electronic medical records nor diagnostic imaging of the included patients in this study. The study population was further screened for hypertension and diabetes mellitus in the NPR registry and the Norwegian Prescription Database [12]. Hypertension and diabetes mellitus were registered as dichotomous variables during the observation period.

#### Data analysis

Statistical analyses were performed with SPSS version 25.0 (IBM Corporation, NY, USA). Descriptive statistics were computed for patient characteristics.

#### Calculations of incidence rates

Incidence rates per 100,000 person-years for patients hospitalized with aSAH were calculated for the adult Norwegian population (3.6–4.0 million inhabitants during the study period), using data from Statistic Norway. Annual decline and confidence intervals for incidence rates were calculated based on the assumption of Poisson distribution.

#### Calculation of case fatality

The time at risk of dying was calculated as the difference between the day of admission and the date of death, and case fatality rates were computed at 30, 90 and 365 days.

#### Predictors of death

We applied a Cox Proportional Hazard regression model to study factors associated with death at 30 days following aSAH. Variables from the univariable analyses with a *p* value < 0.10 were included in the multivariable model. Effect sizes were presented as a hazard ratio with 95% confidence intervals. *P* values < 0.05 were considered statistically significant. Survival analyses of patients with aSAH receiving aneurysm occlusion therapy were analyzed with log-rank tests and visualized using Kaplan-Meier curves.

## Results

In total, 2685 patients identified by NPR were screened for inclusion. We excluded 953 patients based on our exclusion criteria (I60.8 (*n* = 188)) and I60.9 (*n* = 765)), and 1732 patients were included for further analyses.

### Patient characteristics

Patient characteristics are presented in Table 1. Among 1732 included patients, 63% were women. The mean age of males was 58 years and for females 61 years (mean difference 2.7, 95% CI 1,3–4.0, *p* < 0.001). The youngest patient was 18 years while the oldest was 104 years. Arterial hypertension was present in 25% of the included patients and diabetes mellitus among 8%. At the time of aSAH, 21% used oral antithrombotic medication. The majority of the aneurysms (68%) were located in the anterior circulation, compared with 9% in the posterior circulation and 21% in another intracranial location. In total, 64% of the patients underwent aneurysm repair procedures. Among those undergoing aneurysm repair, surgical clipping was performed in 44%, endovascular occlusion in 59%, and 3% underwent both procedures.

**Table 1:** Patient characteristics in 1732 patients with aSAH

Variable	Value
Age, all, years, mean (SD)	60 (14)
Male, years, mean (SD)	58 (14)
Female, years, mean (SD)	61 (14)
Female, *n* (%)	1087 (63)
Oral antithrombotic drugs, *n* (%)	360/1732 (21)
Comorbidities	
Hypertension, *n* (%)	429/1732 (25)
Diabetes mellitus, *n* (%)	133/1732 (8)
Aneurysm location	
Anterior aneurysm (I60.0–I60.3), *n* (%)	1199/1732 (69)
Posterior aneurysm (I60.4–I60.5), n (%)	163/1732 (9)
Other intracranial location (I60.6–I60.7), *n* (%)	370/1732 (21)
Aneurysm repair, *n* (%)	1113/1732 (64)
Clipping, *n* (%)	489/1113 (44)
Coiling, *n* (%)	657/1113 (59)
Coiling and clipping, *n* (%)	33/1113 (3)
CSF diversion procedures, *n* (%)	944/1732 (55)
External ventricular drainage, *n* (%)	890/944 (94)
Shunt, *n* (%)	235/944 (25)
Intracranial pressure monitoring, *n* (%)	543/944 (58)

### Incidence

**Fig. 1:**
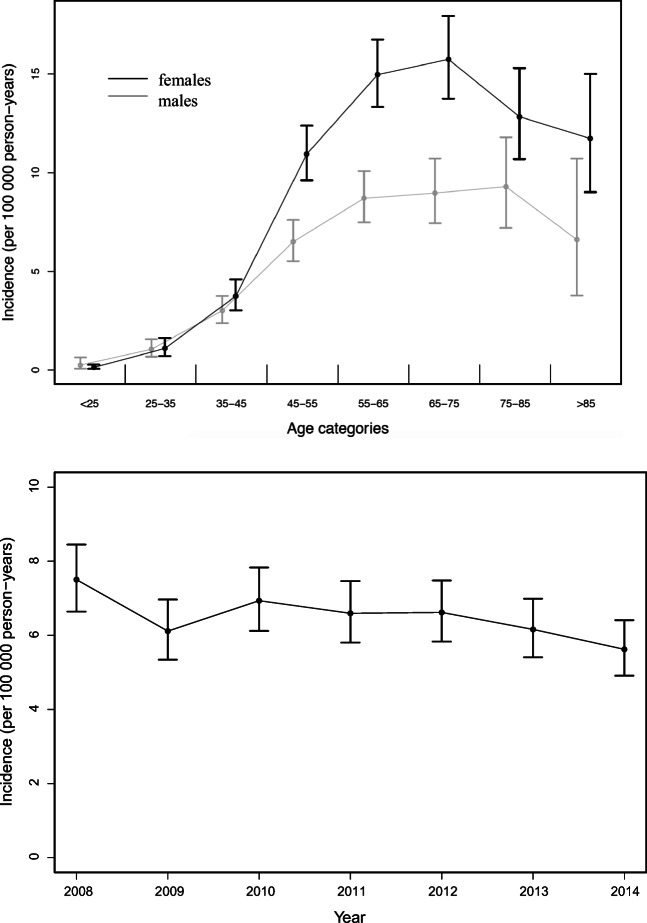
**a** Incidence of aSAH by age and sex. **b** Annual incidence rate from 2008 to 2014

The crude annual incidence rate of aSAH between 2008 and 2014 in Norway was estimated to be 5.7 per 100,000 person-years (95% CI 5.4–6.0). The incidence was higher in females (6.3 per 100,000, 95% CI 5.9–6.7) than for males (4.9 per 100,000, 95% CI 4.5–5.3). The incidence rates of aSAH by age and sex are presented in Fig. 1a and Table 2. The incidence rate increased with age until the sixth decade for both females and males, after which it reached a plateau before it started to decrease from the age of 70. The female-to-male ratio remained relatively equal until the age of 45 years, after which the ratio increased with increasing age to the age of 60 years, and then remained relatively stable. In the study period the annual decline in aSAH incidence was 3.2% per year (*p* = 0.007) (Fig. 1b). In a separate analysis, we included all cases of SAH (I60.0–I60.9) and found a crude annual incidence of 8.8 per 100,000 person-years (CI 8.5–9.2).

**Table 2:** Crude annual incidence rates of aSAH per 100,000 persons between 2008 and 2014 by sex and age group

Age category group	Incidence per 100,000 person-years (95% CI)		
	Female	Male	All
18–25	0.1 (0.1–0.3)	0.3 (0.1–0.6)	0.2 (0.1–0.3)
25–34	1.1 (0.7–1.6)	1.1 (0.7–1.6)	1.1 (0.8–1.4)
35–44	3.7 (3.0–4.6)	3.0 (2.4–3.8)	3.4 (2.9–3.9)
45–54	11.0 (9.6–12.4)	6.5 (5.5–7.6)	8.7 (7.8–9.5)
55–64	15.0 (13.3–16.8)	8.7 (7.5–10.1)	11.8 (10.8–12.9)
65–74	15.8 (13.8–18.0)	9.0 (7.4–10.7)	12.5 (11.2–13.9)
75–85	12.8 (10.7–15.3)	9.3 (7.2–11.8)	11.3 (9.8–13.0)
> 85	11.7 (9.0–15.0)	2.9 (1.7–4.7)	10.1 (8.0–12.6)
Total	6.3 (5.9–6.7)	4.9 (4.5–5.3)	5.7 (5.4–6.0)

### Case fatality

**Fig. 2:**
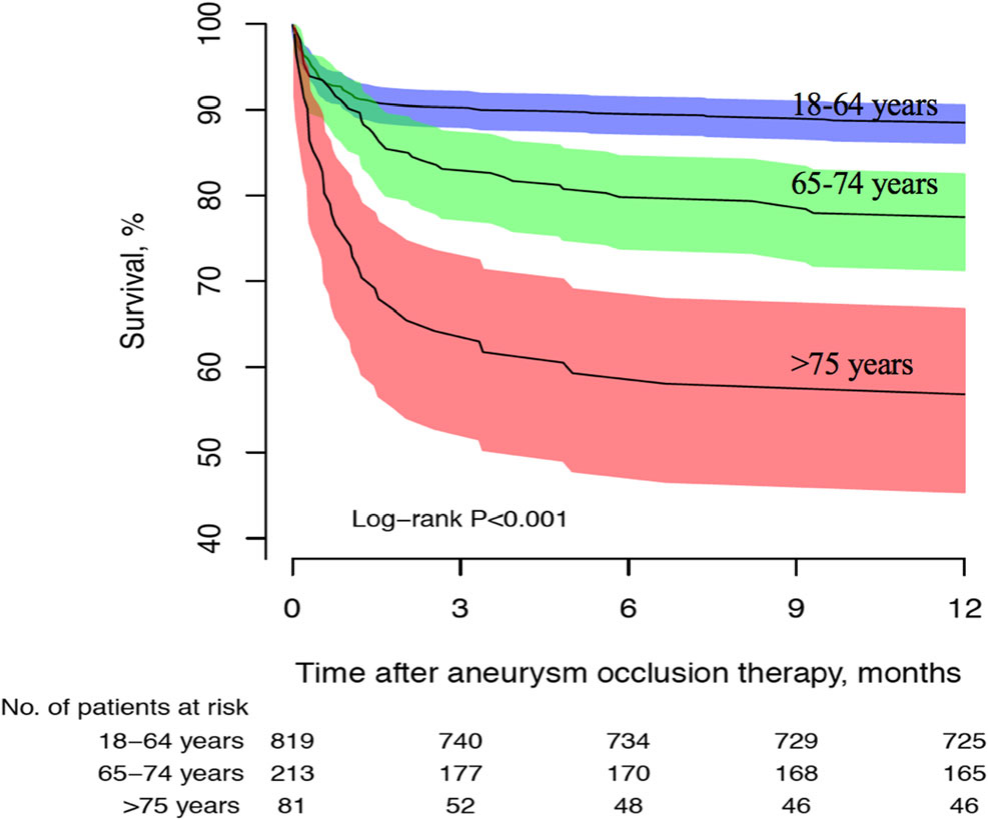
Survival following aSAH in patients receiving aneurysm repair. Error bars represent 95% confidence intervals

Case fatality according to age is presented in Table 3. The cumulative proportions of fatalities at days 30, 90, and 1 year were 22%, 25%, and 37%, respectively. The 30-day case fatality increased with age, from 27% in patients aged < 25 years to 61% in those aged > 85 years of age. The 30-day case fatality incidence remained stable during the study period (0.1% annual increase, *p* = 0.98). Survival for patients with aSAH receiving aneurysm repair is presented in Fig. 2. The mean age of patients receiving surgery was 57 years (SD 12) and 64% were females. Elderly patients (> 75 years) had significantly higher case fatality after surgery compared with younger patients.

**Table 3:** Cumulative case fatality rates (CFR) of aSAH according to age group

CFR (%)	Age category of patients (years)								
	18–25 (*n* = 11)	25–34 (n = 48)	35–44 (*n* = 170)	45–54 (*n* = 403)	55–64 (*n* = 485)	65–74 (*n* = 344)	75–84 (*n* = 192)	>85 (*n* = 79)	All ages (*n* = 1732)
30-day	27	13	10	15	16	24	44	61	22
90-day	27	13	10	17	18	29	50	66	25
365-day	46	21	21	27	32	45	58	82	37

### Predictors of death

In the univariable cox regression analysis the following variables were found to be significantly associated with death 30 days after the aSAH; increasing age (HR 0.4–2.5), hypertension (HR 1.4, 95% CI 1.1–1.7), use of oral antithrombotic drugs at the time of hemorrhage (HR 1.8, 95% CI 1.5–2.3, *p* < 0.001), aneurysms in the posterior circulation (HR 1.6, 95% CI 1.2–2.1, *p* = 0.003), and aneurysm repair (HR 0.2, 95% CI 0.1–0.2, *p* < 0.001) (Table 4). There were no gender differences with respect to survival after aSAH. In the multivariable cox analysis increasing age (HR 0.7–2.2), aneurysm in the posterior circulation (HR 1.7, 95% CI 1.3–2.3, *p* = 0.001), and aneurysm repair (HR 0.2, 95% CI 0.2–0.3, *p* < 0.001) remained associated with death 30 days after aSAH.

**Table 4:** Cox regression analysis for survival at 30 days after aSAH

Variable	Univariable regression		Multivariable regression	
	HR (95% CI)	*p* value	HR (95% CI)	*p* value
Age (categorical)				
18–25 years	Reference group	< 0.001	Reference group	< 0.001
25–34 years	0.4 (0.1–1.7)		0.7 (0.2–2.9)	
35–44 years	0.3 (0.1–1.1)		0.6 (0.2–2.2)	
45–54 years	0.5 (0.2–1.6)		0.9 (0.3–3.0)	
55–64 years	0.5 (0.2–1.7)		1.0 (0.3–3.2)	
65–74 years	0.8 (0.3–2.6)		1.3 (0.4–4.2)	
75–84 years	1.7 (0.5–5.3)		2.0 (0.6–6.3)	
>85 years	2.5 (0.8–8.0)		2.2 (0.7–7.1)	
Sex (female)	1.0 (0.8–1.2)	0.686	–	–
Hypertension	1.4 (1.1–1.7)	0.003	1.0 (0.8–1.2)	0.769
Diabetes mellitus	0.8 (0.5–1.2)	0.285	–	–
Oral antithrombotic medication	1.8 (1.5–2.3)	< 0.001	0.9 (0.7–1.2)	0.485
Aneurysm location (posterior)	1.6 (1.2–2.1)	0.003	1.7 (1.3–2.3)	0.001
Aneurysm repair (any)	0.2 (0.1–0.2)	< 0.001	0.2 (0.2–0.3)	< 0.001

## Discussion

We found a crude annual incidence of aSAH in Norway between 2008 and 2014 of 5.7 per 100,000 person-years. The incidence increased with age until the middle of the sixth decade, after which it remained stable for some years before it started to decrease from the age of 70. We found equal incidence rates for females and males until the age of 45 years, but thereafter we observed higher incidence rates of aSAH in females. We observed a decrease in the annual incidence rate of aSAH in Norway between 2008 and 2014. The case fatality of aSAH is still high, and we observed stable 30-day case fatality incidence during the study period. Increasing age and aneurysms in the posterior circulation were associated with higher 30-day case fatality, whereas aneurysm repair was associated with lower 30-day case fatality following aSAH.

The age and sex distributions found in our study are consistent with prior studies [13, 16, 20]. We found declining incidence in patients aged 70 years and older hospitalized with aSAH. Especially in the elderly, aSAH may be an unrecognized cause of death, and the real incidence of aneurysm rupture probably increases with age beyond 70 years. Unlike other types of strokes, there is a female preponderance for aSAH from the fifth decade of life. The reason for the frequently reported higher incidence of aSAH in females compared with males is unknown, but older age at aSAH event, hormonal factors (including use of hormone replacement therapy) [2, 8], anatomic differences in the circle of Willis [19], and increased vulnerability of smoking, and elevated systolic blood pressure in females compared with males [5, 18, 19] may in part explain the sex gap in aSAH incidence.

The crude incidence of aSAH has previously been estimated to be 9 per 100,000 person-years but varied considerably according to geographic location, age, and sex [2]. Comparisons between studies can be further complicated by different definitions of aSAH, study designs, and case finding procedures [15]. A more recent systematic review from 2010 found a global incidence of aSAH of 6.1 (95% CI 4.9–7.5) per 100,000 person-years, which is more in line with the incidence found in our study [4]. Incidence rates between 7 and 12 per 100,000 person-years have been found in previous studies in Norway [20, 36], Sweden [13, 29, 38], and the US [33]. All of these studies included all ICD-10 coded SAH (I60.0-I60.9) as endpoints, thus complicating comparisons with our study that only included aSAH coded as I60.0-I60.7. When we included all cases of SAH (I60.0-I60.9) we found a crude annual incidence of 8.8 per 100,000 person-years. The low incidence rate found in the present study may depend on our strict criteria for the aSAH diagnosis; nevertheless, we believe that this is a more precise incidence of true aSAH. A Finish study, estimating aSAH coded as ICD-10 I60.0 to I60.6, is more in line with aSAH included in our study. They found a crude annual incidence rate between 6.2 and 10.0 per 100,000 persons between 1997 and 2007 [16].

We estimated an annual decline in aSAH during the study period of 3.2% per year. An annual reduction of SAH incidence has been reported previously in two meta-analyses; the incidence of SAH decreased by 0.6% per year between 1950 and 2005[2], and by 1.7% per year between 1955 and 2014 [4]. Similar trends have been shown in previous epidemiological studies in Norway, [20] Sweden [13], and Finland [16]. However, a decline in aSAH incidence has not been observed in all countries. Stable regional incidence rates have been reported in the US (between 1988 and 2010) [23] and Australia (between 1998 and 2008) [17]. It has been estimated that with effective actions on common lifestyle factors at least half of all strokes may be prevented [7], and thus great effort has been put into reducing cardiovascular risk factors by health education and behavioral change programs. The relatively modest decline in the incidence of aSAH over the past decades compared with that for stroke in general has led to speculations that better control of risk factors is more influential in preventing ischemic stroke than in preventing aSAH [3, 32]. Another explanation might be that the effect of strategies to reduce smoking, one of the most significant risk factors for aSAH, is more delayed in reducing aSAH incidence when compared with interventions that target other modifiable stroke risk factors [6]. Also, the effect of preventive interventions at different times of life might influence the risk of stroke differently, and thus the incidence of different stroke types.

Despite the increasing age of the general population, multiple population-based studies in several countries report a reduction in aSAH case fatality [21, 23, 26, 28, 38]. Our overall 30-day case fatality rate of 22% is lower than the 30-day CFR of 36% that was reported in Norway between 1984 and 2007 [36], and the 28-day CFR of 36.5% and 31.7% that was reported in Sweden between 1985 and 2000 and 1987–2002, respectively [13, 38]. A systematic review published in 2009 (including studies from 1970 to 2008) reported an early case fatality (21 days to 1 month) in low to middle-income countries of 43.9% and in high-income countries 30.0% [6]. Another systematic review from 2009 (including studies from 1972 to 2003) found that the 28-day CFR decreased by 0.8% per year [28]. Declining trends in population-based mortality rates were also reported in Scotland between 1986 and 2005 [24] and England between 1999 and 2010 [26]. In contrast to these studies, we found stable 30-day case fatality incidence during the study period. Existing data describing temporal trends in case fatality for aSAH are limited and conflicting, and comparison between studies is difficult as case finding, diagnostic methods, and management vary substantially between studies. The decreased case fatality observed in studies from the beginning of the year 2000 coincides with the introduction of improved investigative, diagnostic, and treatment strategies for aSAH [10, 25]. We recruited patients with aSAH during a time period when both surgical clipping and endovascular coiling were well-established treatment options in all Norwegian neurosurgical centers, and this might explain both why case fatality is low and remained unchanged during the study period.

We found that older age and aneurysms in the posterior circulation significantly increased case fatality 30 days following aSAH, which is in agreement with previous studies [14, 27, 35]. While smoking, hypertension, and alcohol abuse are established modifiable risk factors for aSAH, the seemingly protective effects of hypercholesterolemia and diabetes in the etiology of aSAH are more uncertain [8, 18, 37]. The decline in incidence between 2008 and 2014 observed in the present study may possibly be attributed to changes in awareness, management, and prevalence of hypertension and diabetes mellitus. According to the Norwegian prescription databse, there was an increase in the number of patients on antihypertensive drugs from 157.1 to 164.4 per 1000 inhabitants in Norway between 2008 and 2014. For antidiabetic drugs, an increase from 29.2 to 33.2 per 1000 inhabitants was observed during the same period [30]. Hypertension and diabetes mellitus were not significantly associated with death 30 days after the hemorrhage in the present study. We found that one-fifth of patients suffering from aSAH were users of oral antithrombotic drugs, and that users of antithrombotic drugs were older than non-users. The use of oral antithrombotic medication was not significantly associated with death 30 days after aSAH. While most studies are consistent in reporting an increased risk of intracranial hemorrhage (including aSAH) in users of anticoagulant drugs compared with no therapy [9, 12], the results are more heterogeneous regarding the risk of aSAH associated with antiplatelet drugs [9, 11]. We could not differentiate between antiplatelet and anticoagulant medications in our study. More than 60% of patients with aSAH underwent aneurysm repair, and occlusion therapy was associated with reduced risk of death 30 days after the hemorrhage. It has been reported previously that treatment of ruptured aneurysms in elderly patients (> 75 years) is feasible, may improve the outcome, and should be strongly considered in patients who are admitted to the hospital in a good condition [27]. Among patients receiving aneurysm repair, we found that elderly patients (> 75 years) had significantly higher case fatality compared with younger patients. Patients receiving surgery are generally younger than those not operated, and a decision is often based on expected better clinical outcome. It is still not settled how coiling compares with clipping in terms of outcomes. The choice of treatment modality depends on several factors including aneurysm location, size, and shape. Whether to clip or coil an aneurysm also depends on neurosurgeons’ and interventional radiologists’ experience and preferences. As aneurysms that are clipped often have different characteristics than those treated with coiling, in addition to the retrospective design, it is difficult to compare the effectiveness of the two treatment modalities in the present study.

### Strengths and limitations

The strengths of this study include the large comprehensive nationwide sample collecting real-world data in a country with a free of charge public health care system that includes all inhabitants. Since the incidence of aSAH can vary between geographical areas, population-based incidence rates from a few hospitals or districts may be misleading. A population-based registry with complete coverage and continuously collected data on all Norwegian residents reduces referral, diagnostic, and information bias. Another strength is that patients were recruited during a time period when both surgical clipping and endovascular coiling were performed in all Norwegian neurosurgical centers. Brain CT scans and ICD-10 classification were also in routine use. The main limitation of this study is the lack of detailed information (i.e., radiological and clinical severity of the aSAH) as we were unable to access patients’ hospital records and diagnostic imaging to validate the diagnoses. In an attempt to decrease the likelihood of reporting inaccurate incidence rates for aSAH, we excluded SAH coded as non-aneurysmal SAH (I60.8-I60.9), and as a consequence, our result may represent an underestimated incidence rate compared with other studies. Although positive predictive values of aSAH in NPR have been found to be high, studies using hospital discharge registries tend to overestimate the incidence rates [31]. We only used the primary diagnosis from NPR for the selection of aSAH episodes. Genuine episodes of aSAH may have had aSAH as a secondary diagnosis leading to underestimation. However, given the severity of symptoms for most instances of aSAH, this source of underestimation should be minimal. Also, the incidence rates reported might represent a slight overestimation as some episodes might be recurrent events that have been incorrectly assessed as first events. Given the low absolute risk of aSAH and the low risk of recurrence, this overestimation will be very small [13]. Another important limitation of our study is that cases dying unexpectedly outside of the hospital from aSAH are not included. The actual cause of death may be hard to determine for persons dying from aSAH before reaching the hospital, especially since the autopsy rate in Norway is the lowest among the Nordic countries [37], and an underestimation of the incidence of aSAH is likely. In previous studies, the share of patients with aSAH that die before arriving at the hospital has been estimated to be 8.3% (range 0.0–21) [16, 28].

## Conclusions

We report a 5.7 per 100,000 person-year crude incidence of aSAH in Norway between 2008 and 2014. Middle and old age females were more prone to aneurysmal rupture than males. In line with previous studies, we observed a decrease in incidence during the study period. Case fatality following aSAH was lower than previously reported, though still high, and we observed an unchanged case fatality rate of aSAH during the study period. Older age and aneurysms in the posterior circulation were associated with a higher risk of death 30 days following aSAH.
